# Species-Specific and Cross-Reactive IgG1 Antibody Binding to Viral Capsid Protein 1 (VP1) Antigens of Human Rhinovirus Species A, B and C

**DOI:** 10.1371/journal.pone.0070552

**Published:** 2013-08-07

**Authors:** Jua Iwasaki, Wendy-Anne Smith, Shane R. Stone, Wayne R. Thomas, Belinda J. Hales

**Affiliations:** 1 Telethon Institute for Child Health Research, Centre for Child Health Research, University of Western Australia, Perth, Australia; 2 School of Paediatrics and Child Health, University of Western Australia, Perth, Australia; Naval Research Laboratory, United States of America

## Abstract

**Background:**

Human rhinoviruses (HRV) are associated with upper and lower respiratory illnesses, including severe infections causing hospitalization in both children and adults. Although the clinical significance of HRV infections is now well established, no detailed investigation of the immune response against HRV has been performed. The purpose of this study was to assess the IgG1 antibody response to the three known HRV species, HRV-A, -B and -C in healthy subjects.

**Methods:**

Recombinant polypeptides of viral capsid protein 1 (VP1) from two genotypes of HRV-A, -B and -C were expressed as glutathione S-transferase (GST) fusion proteins and purified by affinity and then size exclusion chromatography. The presence of secondary structures similar to the natural antigens was verified by circular dichroism analysis. Total and species-specific IgG1 measurements were quantitated by immunoassays and immunoabsorption using sera from 63 healthy adults.

**Results:**

Most adult sera reacted with the HRV VP1 antigens, at high titres. As expected, strong cross-reactivity between HRV genotypes of the same species was found. A high degree of cross-reactivity between different HRV species was also evident, particularly between HRV-A and HRV-C. Immunoabsorption studies revealed HRV-C specific titres were markedly and significantly lower than the HRV-A and HRV-B specific titres (*P*<0.0001). A truncated construct of HRV-C VP1 showed greater specificity in detecting anti-HRV-C antibodies.

**Conclusions:**

High titres of IgG1 antibody were bound by the VP1 capsid proteins of HRV-A, -B and -C, but for the majority of people, a large proportion of the antibody to HRV-C was cross-reactive, especially to HRV-A. The improved specificity found for the truncated HRV-C VP1 indicates species-specific and cross-reactive regions could be defined.

## Introduction

Human rhinovirus (HRV) infection is not only the main cause of the common cold [1] but is strongly associated with exacerbations of asthma, acute bronchitis, chronic obstructive pulmonary disease in adults, as well as pneumonia in immunocompromised patients [2]–[4]. The biologically and molecularly distinct HRV-C species, which has been found in hospitalised patients, has attracted attention for a role in asthma exacerbation of children [5] and for serious disease in adults and the elderly, including transplant patients [4]. HRV-C has been difficult to grow in vitro [6], so its detection has relied on the sequencing of viral RNA in tissue samples, the method now most commonly used to detect HRV-A and HRV-B. The prevalence of RNA detection for all species has been higher in hospitalized patients [3] but has also been high in healthy subjects often in similar abundance [7].

Neutralising antibodies provided the key observations that first distinguished HRV-A from HRV-B but, because they are directed to a limited number of capsid structures that frequently mutate [8], they have limited application for sero-epidemiology and profiling the history of immune responses to repeated infections. The neutralising antibodies induced by experimental HRV inoculation appear 2–3 weeks after infection, well after the clearance of virus and resolution of the symptoms [9], [10], and at least for experimental infection can no longer be detected after 9 months [11]. As found for other respiratory virus infections, it is likely that innate immunity and T-cell responses are the most important responses for the resolution of infection, while neutralising antibodies protect against short-term reinfection [12]. Antibodies have been shown to interact with T-cells in adaptive immune responses to clear viruses, but interestingly these were non-neutralising antibodies [13], [14] that are also likely to be more abundant. Such interaction between non-neutralising antibodies and T-cells might also be expected to affect immunopathology, for example in aiding the capture of virus by monocytes and thereby enhancing inflammatory cytokine production [15]–[17].

The objectives of this study were to use antibody-binding to defined HRV antigens to determine whether immune responses to the different HRV species could be distinguished, and further, if the antibody responses to the different species had different characteristics. The antigen examined was the VP1 capsid protein that exhibits the highest surface exposure of the four HRV capsid proteins and forms part of neutralising antigenic determinants [18]. It, along with VP2 and VP3, exhibits the amino acid sequence variation found amongst genotypes of each species. Comparing the VP1 of HRV-A and HRV-B, most isolates form a cluster with 58–99% amino acid sequence identity within each species, contrasting with the 35–44% identity found between HRV-A and HRV-B [19]. Similarly, the VP1 of HRV-C genotypes has 59–90% amino acid sequence identity within HRV-C, and about 45% and 36% identity between HRV-C and HRV-A and HRV-B respectively [2]. Our studies here show high IgG1 antibody binding to recombinant full length VP1 antigen constructs for all species, with high titres of species-specific antibody for HRV-A and HRV-B, in contrast to a low prevalence and titre for HRV-C. A construct of HRV-C made without the N-terminal alpha-helical domain showed a lower degree of antibody binding but, significantly, was restricted to sera from subjects with species-specific antibodies.

## Materials and Methods

### Study Population

Sera from 63 adults with no evidence of current respiratory illness from the general population in Perth, Western Australia were examined (40 women and 23 men; age ranged from 20–53; median age 31).

### Ethics Statement

Written informed consent was obtained from all participants. Ethics approval was provided by The Princess Margaret Hospital Human Ethics Committee.

### HRV Antigens

VP1 antigens from two genotypes of HRV-A, -B and -C were produced in *Escherichia coli (E.coli)* as they represent genetically disparate variants within each species. The following HRV VP1 proteins were produced: HRV-A34 (GenBank accession number FJ445189.1) and HRV-A1B (D00239.1) of HRV-A species; HRV-B14 (NC001490) and HRV-B69 (FJ445151) of HRV-B species; and HRV-C3 (EF186077 [20]) and HRV-C5 (EF582386 [2]) of HRV-C species (Table S1). The VP1 of another enterovirus, human poliovirus (HPV) Sabin VP1 (AY184219.1) was produced as a control to determine specificity in antibody binding to HRV. The amino acid sequence identities of the VP1 proteins are shown in Table 1.

**Table 1:** Amino acid sequence identity for six HRV VP1 and HPV Sabin 1 VP1.

		HRV-A		HRV-B		HRV-C		HPV
		34	1B	14	69	3	5	Sabin
**HRV-A**	**34**		77.7	35.2	35.5	41.4	44.6	35.2
	**1B**			36.9	37.2	39.4	43.6	33.9
**HRV-B**	**14**				71.1	32.9	33.1	40.5
	**69**					33.8	36.2	42.2
**HRV-C**	**3**						61.9	28.3
	**5**							32.0
**HPV**	**Sabin**							

### Expression and Purification of Recombinant HRV VP1

The nucleotide sequences encoding VP1 cDNAs were synthesized with codon optimization for expression in *E.coli* by GenScript (Piscataway, NJ). They were subsequently engineered for expression as fusion proteins with glutathione S-transferase (GST) at the N-terminus and a hexa-histidine tag on the C-terminus. The genes were amplified by PCR from cDNA in pUC57 as a template. Specific PCR primers were designed to amplify the VP1 coding sequence and the addition of six histidine residues. PCR was performed using high-fidelity *Pfu* DNA polymerase (Promega, Madison, WI) using the following conditions: 1 cycle at 95°C for 5 min; 35 cycles at 95°C for 1 min, 55°C for 30 s, and 74°C for 3 min; and finally 74°C for 7 min. The PCR products were extracted from a 1% agarose gel using the Gel Purification Kit (Qiagen, Hilden, Germany). The amplified DNA fragment was digested with *Bam*HI and *Eco*RI, and ligated into a *Bam*HI/*Eco*RI digested pGEX-2T expression vector (GE Healthcare). After transformation into chemically competent TOP 10 *E.coli*, positive clones were verified by DNA sequencing (AGRF, Australia). The recombinant plasmid was subsequently transformed into the *E.coli* expression strain BL21. A GST control was produced directly from pGEX-2T.

For expression of VP1, an overnight culture diluted 1∶20 was grown to OD_600 nm_ 0.6 and induced with 0.1 mM IPTG at 30°C for 2 hours. The *E.coli* pellets were resuspended in 5 ml/g Buffer A (150 mM NaCl, 50 mM NaH_2_PO_4_, 1% Tween-20, 1 mM PMSF, pH 8) with the addition of lysozyme (1 mg/ml, Sigma-Aldrich, St Louis, MO), sonicated and clarified at 18,000 rpm for 60 min. The soluble supernatant was then purified in accordance with the manufacturer’s protocols (Sigma, USA) with modifications. Briefly, glutathione agarose was pre-equilibrated with Buffer B (150 mM NaCl, 50 mM NaH_2_PO_4_, 0.1% Tween-20, pH 8). The clarified lysate was bound to the matrix and the column was washed with 10×column volume with Buffer B. Bound protein was eluted with Buffer C (Buffer B +10 mM reduced glutathione). Fractions collected from the column containing recombinant protein were pooled, concentrated and passed over a high resolution S300 26/60 column (GE Healthcare, Uppsala, Sweden).

The purity of the recombinant proteins were analysed by size exclusion chromatography and SDS-PAGE analysis using a 12.5% electrophoretic gel and GelCode Blue Safe Protein Stain (Thermo Scientific). Protein concentrations were calculated using OD_280 nm_ and extinction coefficients calculated for each fusion protein.

### Circular Dichroism Analysis

Purified protein preparations were diluted to a final concentration of 3 mM in 10 mM potassium phosphate, 100 mM (NH_4_)_2_SO_4_ buffer (pH 8) and circular dichroism (CD) spectroscopy performed as outlined in Hales *et al.* [21]. Briefly, CD spectral measurements were performed on an OLIS DSM-1000 spectrophotometer over the wavelength range 260–190 nm operating at 20°C in a 1-mm path length cell (Starna). The analysis of protein secondary structure from CD spectra was performed using the DiChroWeb Server [22] and the CDSSTR algorithm and reference sets 4, 7 and SP175 (short) [23].

### Immunoassays to Determine Total IgG1 Antibody Binding

The DELFIA® assay is described in detail elsewhere [24] and was performed with modifications. In brief, microtitre plates (Nunc, Roskilde, Denmark) were coated with 100 µL of purified VP1 antigens diluted to 0.125–4 µg/ml in 50 mM Na_2_CO_3_/NaHCO_3_ (pH 9.6) at 4°C overnight. The concentration of antigen used for coating was standardised by titrating the coating concentrations using monoclonal anti-glutathione-S-transferase antibody (Sigma-Aldrich, St Louis, MO) to measure the amount adhering to the well. Optimal concentrations were determined by referencing to a recombinant GST-fusion Der p 2 standard that was later used for quantitation of antibody binding (see below). Each test sera was diluted to a final concentration of 1∶100 in blocking buffer (0.5% bovine serum albumin in 50 mM Tris-HCl, 0.9% NaCl, 0.05% sodium azide (pH 7.4) buffer with 0.01% Tween-20) and incubated overnight at 4°C with shaking. The coated plates were washed 5 times with 50 mM Tris-HCl, 0.9% NaCl (pH 7) buffer with 0.01% Tween-20. This washing step was conducted after each incubation step. The plates were blocked with 200 µL of blocking buffer for 2 hours at room temperature with shaking. 100 µL of sera was then added to the antigen-coated wells and incubated for 2 hours at room temperature with shaking. The wells were then incubated with 100 µL of biotinylated anti-human IgG1 (BD Pharmingen, USA) diluted 1∶2000 in assay buffer (Wallac, Oy) for 2 hours at room temperature with shaking. This was then followed by incubation for 30 minutes with 100 µL Europium-labelled streptavidin diluted 1∶1000 in assay buffer, with shaking. The final washing step involved washing the plates 8 times before the addition of 100 µL enhancement solution. The plates were read using a Wallac Victor 3 plate reader (Wallac, Oy).

### Immunoabsorption Assays to Determine Species-Specific IgG1 Antibody Binding

Immunoabsorption assays were conducted to determine the species-specific IgG1 antibody binding and were performed as per the immunoassay above, except that each test sera was pre-incubated in a lysate mixture of *E.coli* producing the other two HRV species and HPV Sabin VP1 to absorb out cross-reactive binding. The lysates (produced using soluble supernatants following sonication as described above) were used at a final concentration of 1∶250 shown by pilot experiments to be an excess amount to ensure complete inhibition where present.

### Quantitation of IgG1 Antibody Binding

A titration of reference sera was included on every plate to construct a standard curve and act as a positive control to assess reproducibility. The correlation of variation was less than 5% between plates. The standard curve was then used to quantitate the IgG1 binding to the VP1 antigens. Human myeloma IgG1 (Sigma-Aldrich, St Louis, MO) was used as a negative control to determine non-specific binding and the identification of negative control sera (Figure S1). Three negative control sera for each antigen were included on every plate. The absolute quantity of antigen-specific IgG1 (ng/ml) in the reference sera was interpolated from a titration curve constructed with equivalent concentrations of recombinant GST-fusion Der p 2 and a standardised humanised anti-Der p 2 chimeric IgG1 (Indoor Biotechnologies, Charlottesville, USA) [25], [26]. Wells coated with the same quantity of antigen were used, as determined by the binding of anti-glutathione-S-transferase antibody. The lower limit of detection for IgG1 binding was 500 ng/ml and thus negative values were assigned 50% of the lower limit of detection (250 ng/ml).

### IgG1 Competitive Inhibition Studies

Sera from individuals with high IgG1 binding to two HRV species were diluted 1∶50 with blocking buffer and then mixed 1∶2 with different dilutions of purified VP1 antigens to give final concentrations ranging from 0.004 to 8 µg/ml of the inhibiting protein. Following overnight incubation at 4°C, the mixtures were centrifuged at 14,000 *g* for 10 min and 100 µL aliquots of the supernatant were added to the antigen-coated plates for the DELFIA® assays. The assay was then developed as per the IgG1 immunoassay. An irrelevant recombinant antigen, either Fel d 3 or the bacterial protein pneumococcal surface protein (Psp)-C, was used as a negative control to illustrate that any cross-reactivity that existed was specific to HRV species and, where present to HPV Sabin. Recombinant Fel d 3 and Psp-C were both expressed in an *E.coli* expression system and purified by multiple steps of purification. Details of the antigen preparations have been described previously [24], [27].

### Statistical Analysis

The absolute antibody binding to each VP1 antigen was log-transformed to approximate a normal distribution. Differences in antibody binding to different antigens were compared by the paired *t*-test. A *P* value <0.05 was considered significant. The geometric mean and 95% confidence intervals were calculated. Correlations between antibody responses were analysed by the Spearman correlation. All analyses were performed using GraphPad Prism Software (La Jolla, USA).

## Results

### Expression of Recombinant GST-fusion HRV VP1 Antigens

**Figure 1:**
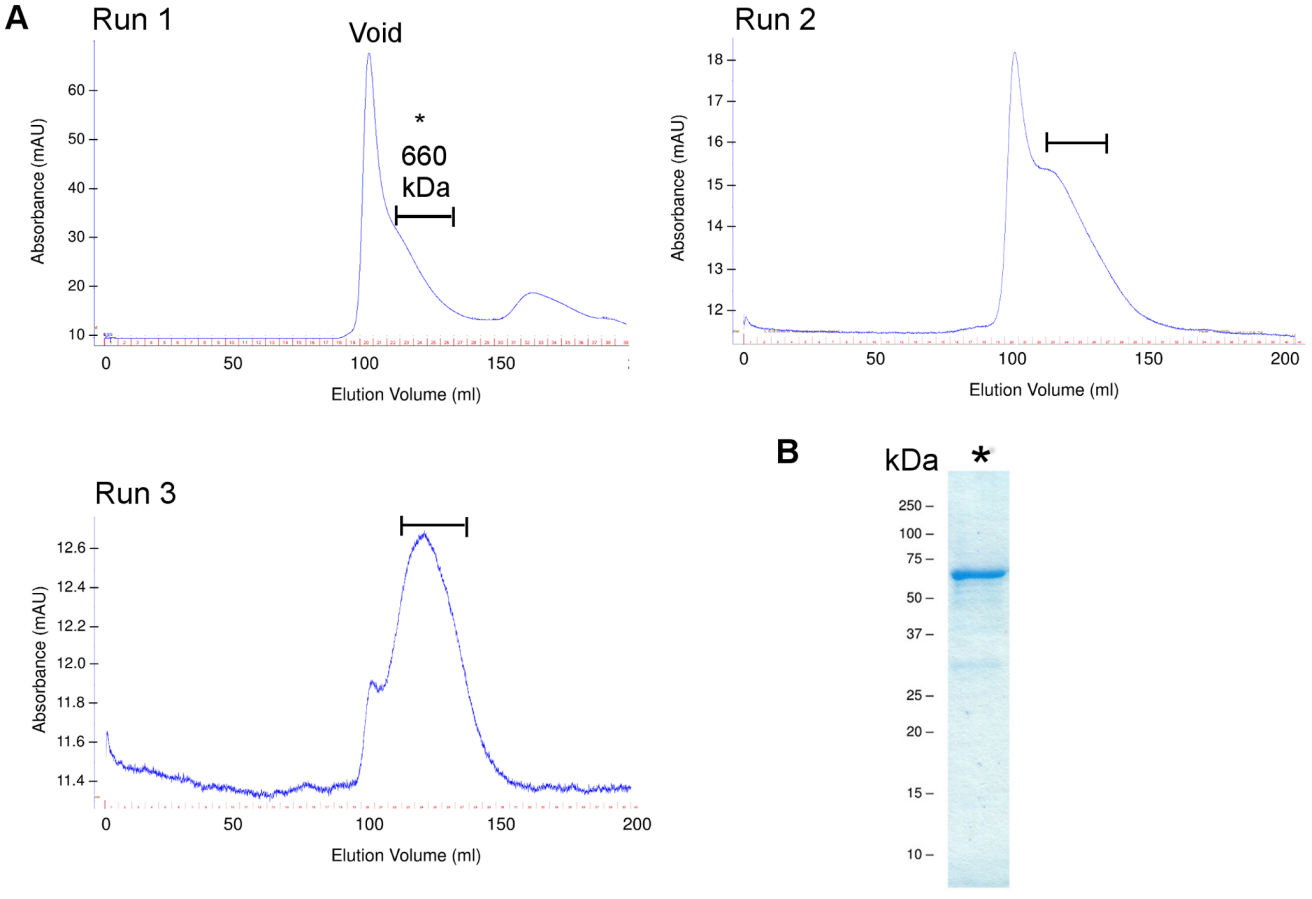
Purification and SDS-PAGE analysis of recombinant HRV-C3 VP1 pGEX-2T. (A) Chromatograph of HRS300 size exclusion chromatography immediately following affinity chromatography (Run 1). The void volume and the peak at 660 kDa are indicated above each of the main peaks. Each fraction has a 5 ml elution volume. Fractions containing the 660 kDa peak from Run 1 were pooled and concentrated for a second run and third run on the HRS300. The bar indicates fractions used for subsequent runs and for binding analysis. (B) A 10 µL aliquot of the pooled fractions containing the 660 kDa peak collected from the first run of gel filtration (as indicated by *) was analysed by SDS-PAGE. The MW of the protein standard is indicated.

Recombinant GST-fusion HRV and HPV Sabin VP1 with a C-terminal histidine tag was produced in *E.coli* as a soluble protein. Following glutathione-agarose affinity chromatography and sequential gel exclusion chromatography, the final gel filtration isolated a multimeric protein estimated to be 660 kDa (Figure 1A) distinct from the aggregated protein that was excluded from the gel. Under denaturing conditions, the SDS-PAGE revealed a single band of 59 kDa (Figure 1B).

### Structural Characterization of HRV VP1 Antigens

Purified GST-fusion proteins were subjected to subtractive CD analysis in which the signal from the GST control was subtracted from the fusion proteins. The CD spectrum of the GST dimer showed predominantly α-helical structure analogous to published data [28]. The CD spectra of HRV-A34, HRV-B14 and HRV-C3 VP1 demonstrated the presence of well-folded secondary structure, with broad minima between 208–220 nm indicative of mixed β-sheet and α-helical structure. Minima at 194 nm, close to the expected 198 nm minimum, also suggested random coil conformation (Figure S2). Secondary structure calculations of HRV-A34 VP1 using the CDSSTR algorithm and reference set 4 indicated the presence of 10% α-helix and 30% β-sheet content (Table 2), comparable to the published secondary structure of HRV-A1A VP1 ([29]; pdb: 1R1A). The percentages of secondary structure of recombinant HRV-B14 VP1 was also similar to published data on native HRV-B14 VP1 ([30]; pdb: 1NCQ).

**Table 2:** Percentages of the secondary structure of the circular dichroism (CD) spectra of HRV VP1 antigens following subtraction of GST*.

	α-helix	β-sheet	β-turns	Disordered
**HRV-A34 VP1**	10	30	24	36
**HRV-B14 VP1**	10	36	25	27
**HRV-C3VP1**	13	33	25	29
**Truncated** **HRV-C3 VP1**	12	33	26	30

* Analysed using DiChroWeb Server: CDSSTR algorithm, reference set 4.

### Total IgG1 Antibody Binding to HRV VP1 Antigens

**Figure 2:**
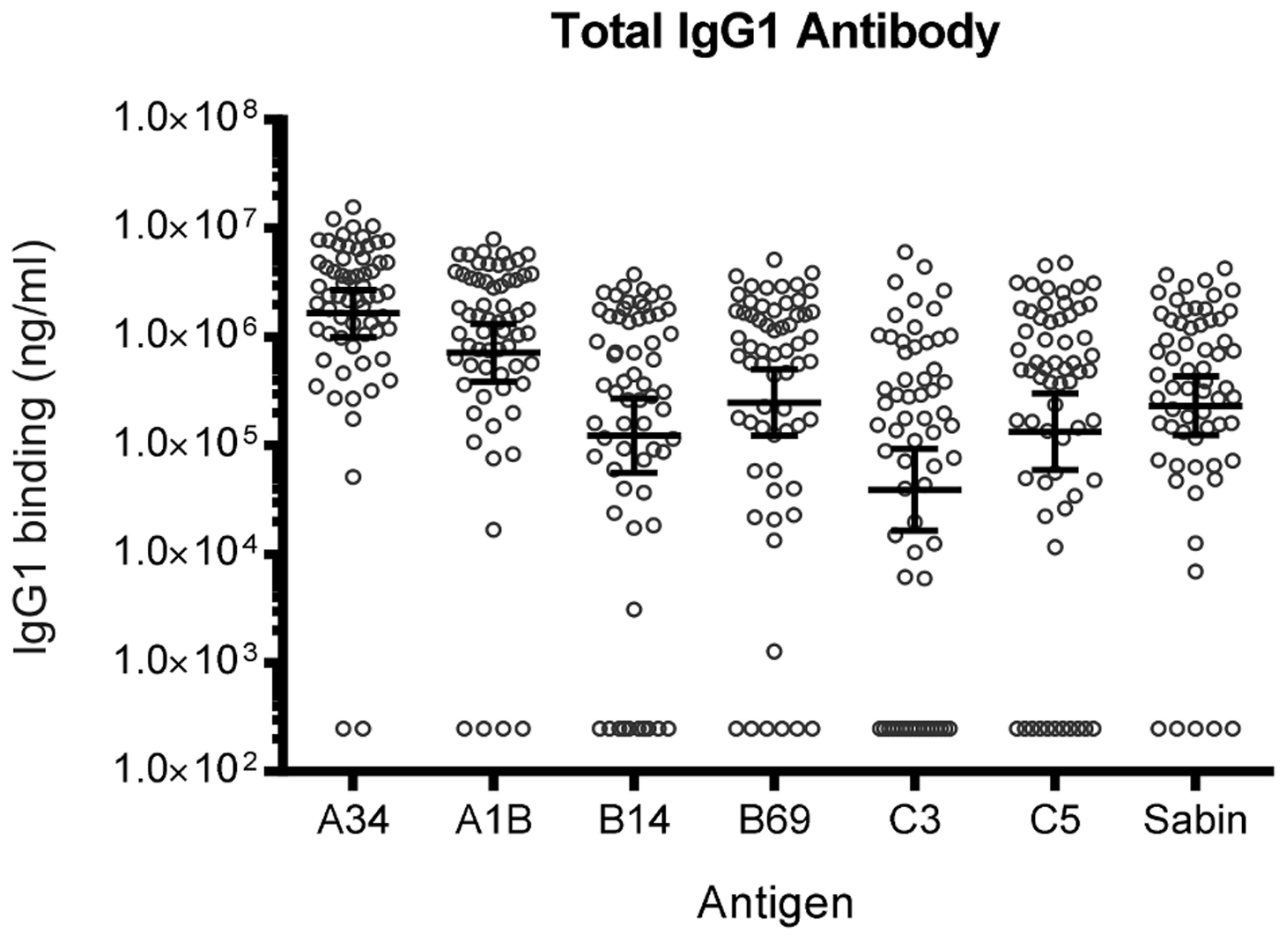
Total IgG1 antibody binding (ng/ml) to HRV and HPV Sabin VP1 antigens in 63 healthy adults. The geometric mean and 95% confidence interval are indicated. Human myeloma IgG1 was used as a negative control to determine non-specific binding and identification of negative control sera. Three negative control sera for each antigen and a titration of reference sera were included on every plate for the quantitation of IgG1 binding and to assess reproducibility of the assay.

Initially, total IgG1 antibody binding to each of the HRV VP1 antigens was measured. This provided a measure of IgG1 binding to each of the species, including any reactivity that may be directed to cross-reacting epitopes shared with other HRV species, or with other enteroviruses. The VP1 antigens bound IgG1 antibodies from the 63 adult sera at high titres (Figure 2). There was no significant difference between HRV-A, -B, -C or HPV Sabin VP1 titres. However, the prevalence of total IgG1 reactivity was higher for HRV-A variants (97% for HRV-A34 and 94% for HRV-A1B) than HRV-B (83% for HRV-B14 and 89% for HRV-B69) and HRV-C (70% for HRV-C3 and 79% for HRV-C5).

### Competitive Inhibition Studies

**Figure 3:**
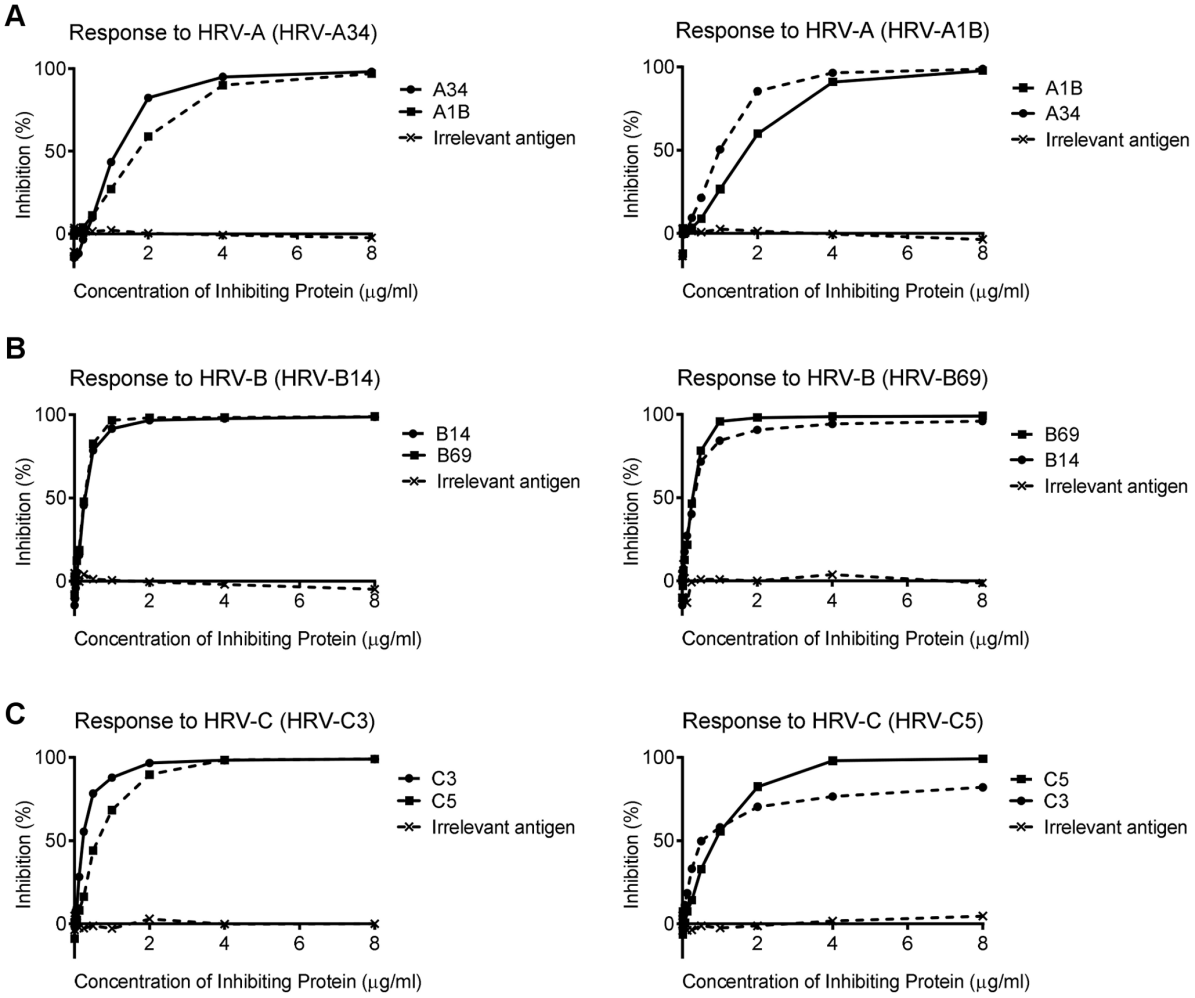
Strong IgG1 cross-reactivity between two HRV genotypes of the same species. Each graph represents percentage of inhibition in individual sera. Subjects that had high total IgG1 titres to testing antigens were selected for competitive inhibition assays and example inhibition assays are shown. (A) Level of inhibition between HRV-A34 and HRV-A1B (HRV-A species). (B) Level of inhibition between HRV-B14 and HRV-B69 (HRV-B species). (C) Level of inhibition between HRV-C3 and HRV-C5 (HRV-C species). The inhibition of binding by an irrelevant recombinant antigen (Fel d 3), used as a negative control, are also indicated.

To examine cross-reactivity between the genotypes, the cross inhibition of the two VP1 proteins representing each species was examined. Sera from two high responder subjects were selected for each species and reciprocal competitive inhibition assays conducted with antigen from the two genotypes for each species. The genotype VP1 for the HRV-A strongly cross inhibited each other (Figure 3A) as did the genotypes for HRV-B (Figure 3B). The inhibition for HRV-C was similar, although the inhibition of binding to HRV-C5 by HRV-C3 plateaued at 80% (Figure 3C). The inhibitions all reached a maximum with antigen concentration of 4 µg/ml or less.

**Figure 4:**
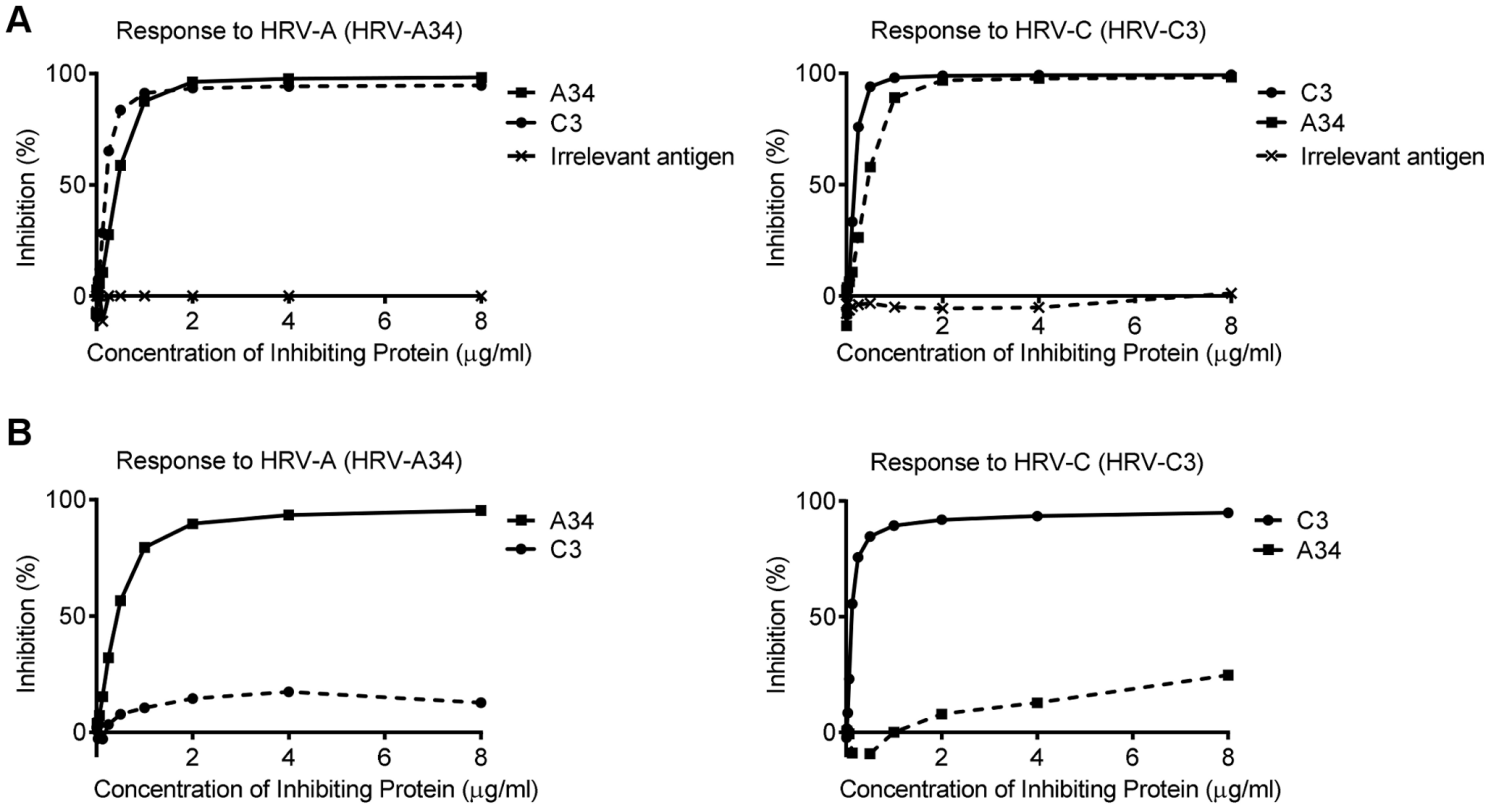
Cross-reactivity between HRV-A and HRV-C. (A) An example of strong cross-inhibition in HRVA34 and HRV-C3 double-positive sera. (B) An example of poor cross-inhibition between HRV-A34 and HRV-C3 observed in a minority of subjects who had high IgG1 titres to both HRV-A and HRV-C. The inhibition of binding to HRV-A34 and HRV-C3 by an irrelevant recombinant antigen (bacterial protein, Psp-C), used as a negative control, are also indicated.

**Figure 5:**
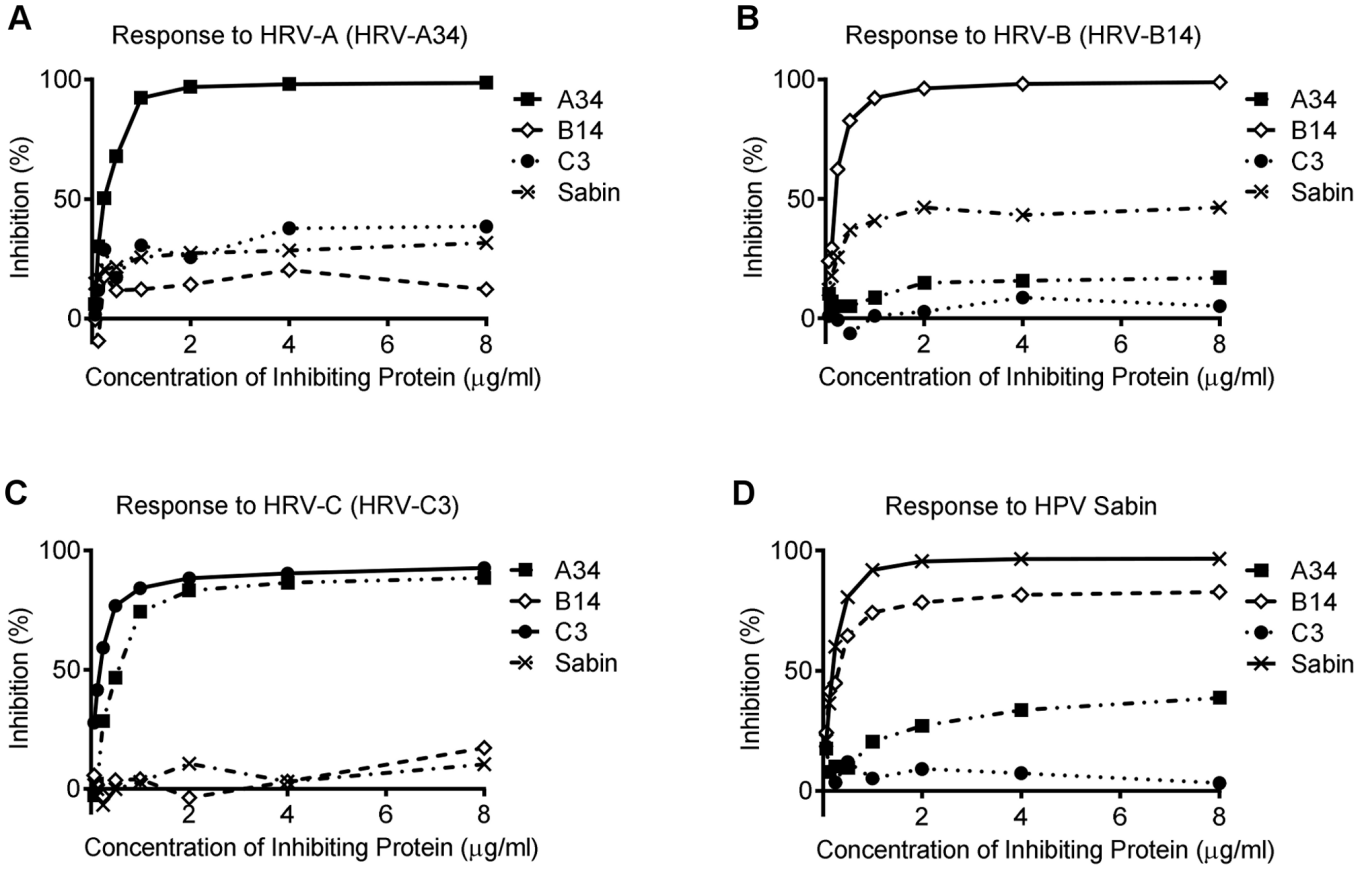
An example of the level of cross-reactivity found between HRV species and HPV Sabin VP1 in an individual’s serum. (A) HRV-A VP1 antigen inhibited by HRV-B, HRV-C and HPV Sabin antigens. (B) HRV-B VP1 antigen inhibited by HRV-A, HRV-C and HPV Sabin antigens. (C) HRV-C VP1 antigen inhibited by HRV-A, HRV-B and HPV Sabin antigens. (D) HPV Sabin antigen inhibited by antigens representing the three HRV species. An irrelevant recombinant antigen (Fel d 3) was used as a negative control for each competitive inhibition assay (data not shown).

Cross inhibition measured with VP1 between the species had a high degree of variation with different sera. The reciprocal inhibition with the serum shown in Figure 4A shows almost 100% reciprocal inhibition between HRV-A34 genotype antigen of HRV-A and the HRV-C3 genotype of HRV-C, while the inhibition between HRV-A and HRV-C shown in Figure 4B in another subject, was low. Inhibition of IgG1 binding to HRV VP1 was negligible using the bacterial protein, Psp-C as a control (Figure 4), and HRV VP1 was similarly unable to inhibit IgG1 binding to Psp-C. Further competitive inhibitions as exemplified for the individual in Figure 5 showed there were also lesser inhibitions between all the VP1 including with HRV-B and HPV Sabin. The binding to HPV Sabin was strongly cross inhibited by the HRV-B14 genotype of HRV-B, while inhibition by HRV-A and HRV-C was minimal (Figure 5D). The pattern, however, was variable when other subjects were examined.

**Figure 6:**
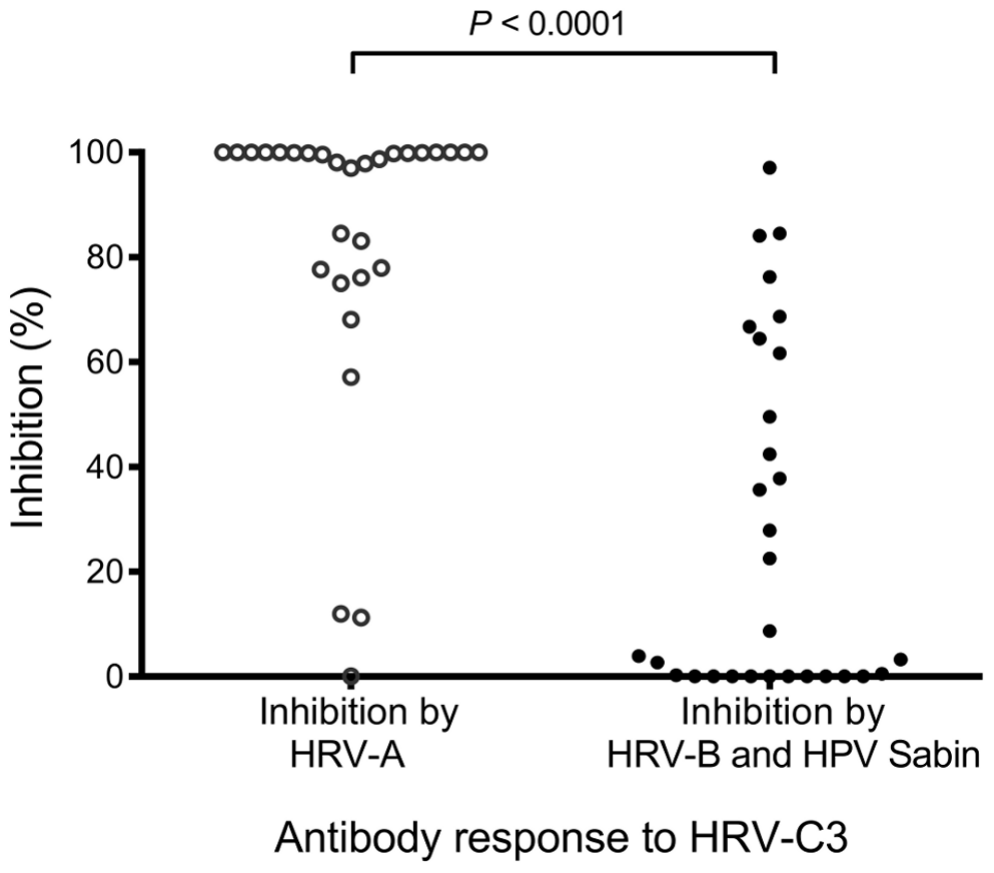
Inhibition of IgG1 binding to HRV-C3 VP1 by HRV-A or HRV-B and HPV Sabin. Inhibition by lysate containing HRV-A, or a mixture of HRV-B and HPV Sabin in 30 subjects with high total IgG1 titres to HRV-C3. Percentage of cross-reactivity to the inhibiting species was calculated by (Titre after absorption/Total IgG1)×100. *P*<0.0001 between inhibition by HRV-A and inhibition by HRV-B and HPV Sabin. Three negative control sera and a titration of reference sera were included on every plate to assess reproducibility and quantitation of IgG1 binding.

Despite the variation, absorption assays using sera of 30 subjects who had high IgG1 titres to HRV-C (HRV-C3) revealed a much greater inhibition of antibody binding to HRV-C with HRV-A, than with HRV-B or HPV Sabin. Here, absorption with HRV-A lysate resulted in complete inhibition of binding to HRV-C3 in the majority of subjects, showing frequent high cross-reactivity (Figure 6). In comparison, absorption with a mixture of HRV-B and HPV Sabin lysates produced lower and less frequent inhibition.

### Species-specific IgG1 Antibody Binding to HRV VP1 Antigens

**Figure 7:**
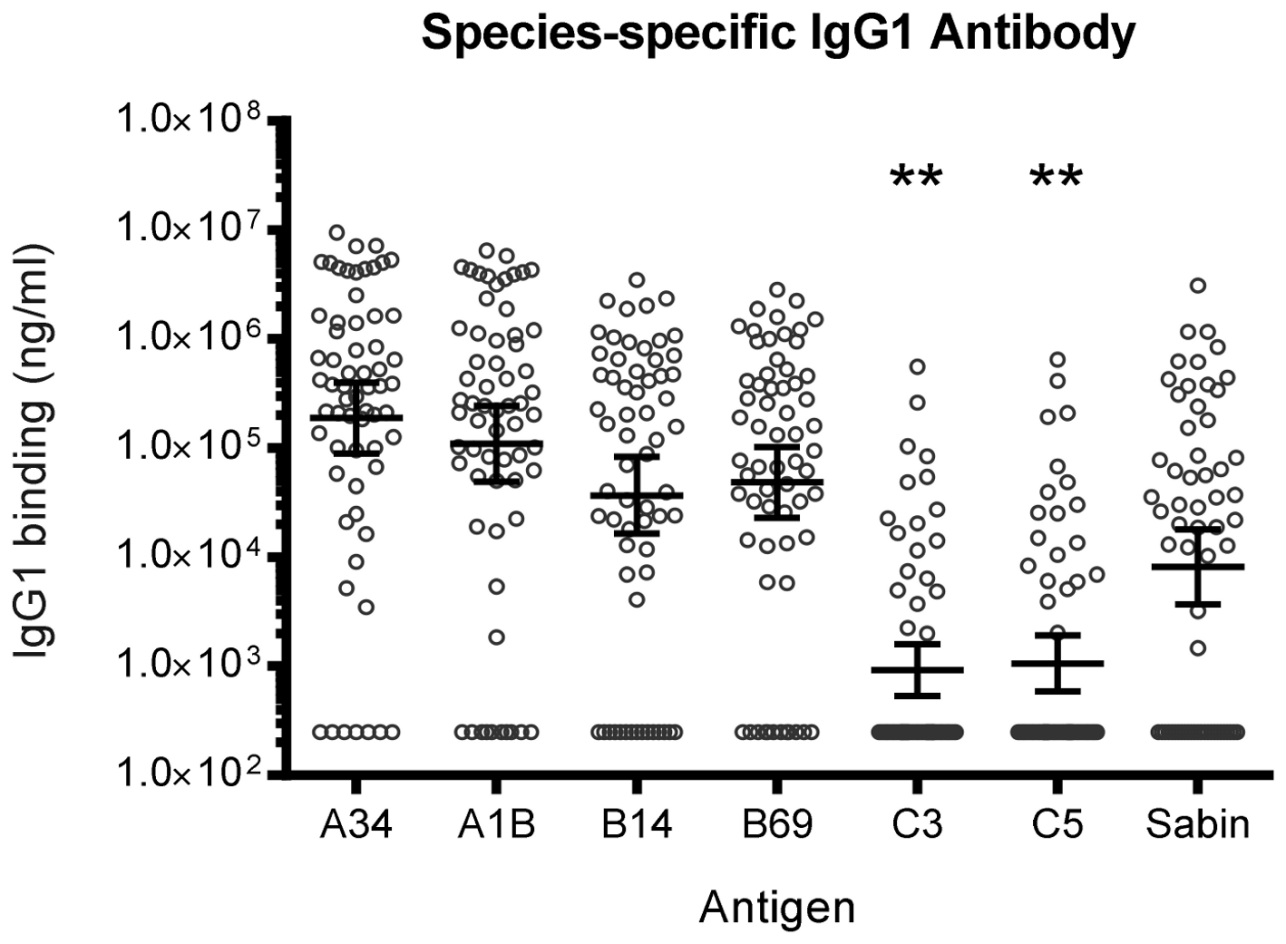
Species-specific IgG1 antibody binding (ng/ml) to HRV and HPV Sabin VP1 antigens in 63 healthy adults. Each sera was absorbed with lysates containing the VP1 of the other HRV species, as well as HPV Sabin VP1. IgG1 binding to HRV-C (HRV-C3 and HRV-C5) antigens were significantly lower than HRV-A, HRV-B and HPV Sabin titres (*P*<0.0001) as indicated by **. Human myeloma IgG1 was used as a negative control to determine non-specific binding and identification of negative control sera. Three negative control sera for each antigen and a titration of reference sera were included on every plate for the quantitation of IgG1 binding and to assess reproducibility of the assay.

**Figure 8:**
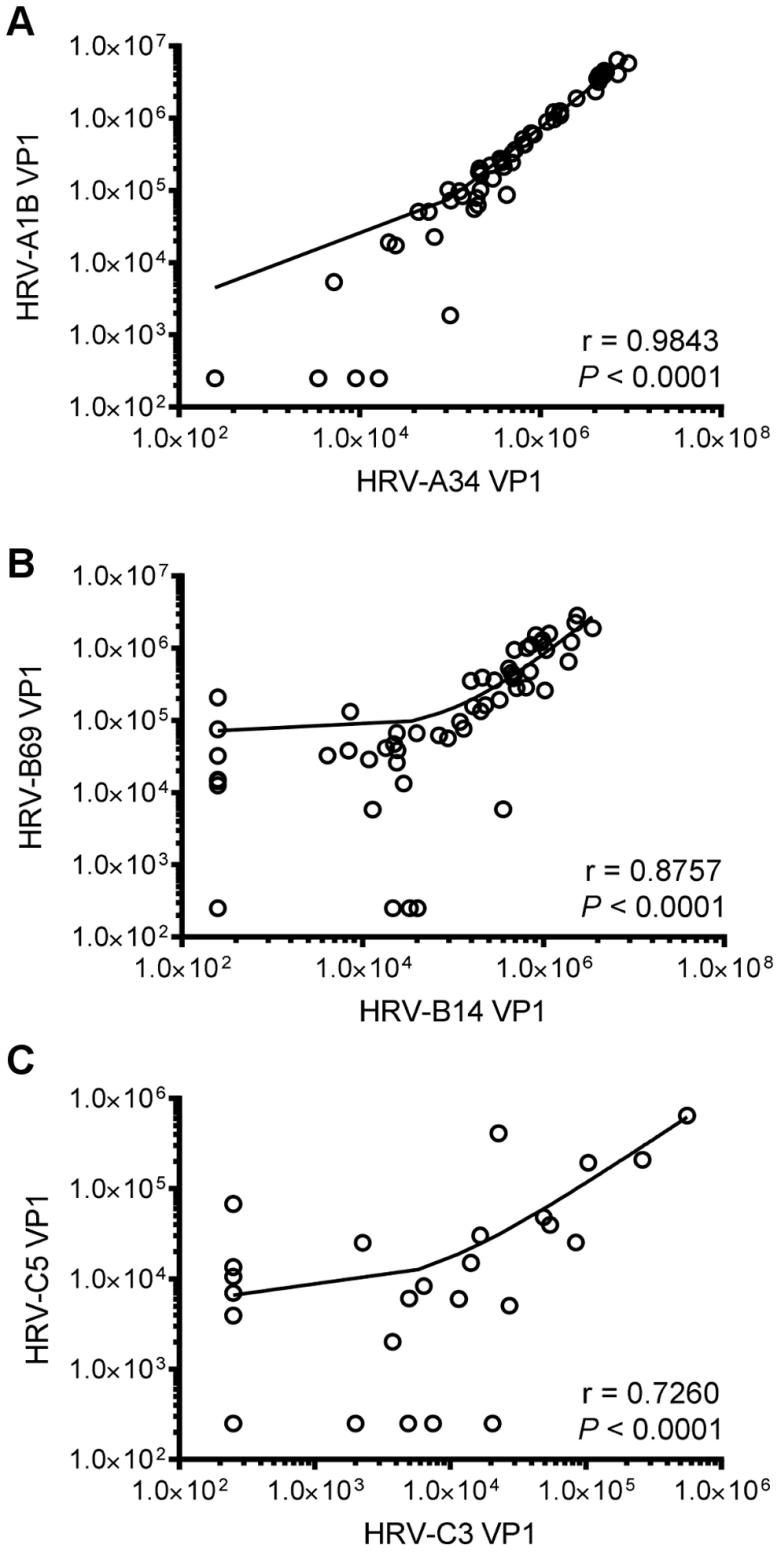
Intra-species correlation of IgG1 responses in 63 adult subjects. Log correlation of IgG1 binding (ng/ml) between two genotypes of (A) HRV-A (B) HRV-B and (C) HRV-C.

In the light of the competitive inhibition studies, the species-specific titres were determined following absorption of each sera with lysates containing the VP1 of the other species. Thus antibodies to the HRV-C genotypes were measured after absorption with a mixture of HRV-A, HRV-B and HPV Sabin. The same procedure was used for the antigens from the other species. Each assay included three negative control sera, as determined by human myeloma IgG1, and reference sera. The species-specific titres determined, for each of the two genotypes of HRV-A, HRV-B and HRV-C, show the very high titres of antibody to the HRV-A species compared to usually low titres for HRV-C, and much higher prevalence of the species-specific binding antibodies to HRV-A than HRV-C (Figure 7; *P*<0.0001). Only 19/63 subjects had antibodies specifically attributable to HRV-C (HRV-C3) and the titres of the responders did not reach the titres found for HRV-A. The HRV-B titres were similar in size and prevalence to the HRV-A. The correlation of the antibody binding between the VP1 antigens of each genotype within each species was very high (Figure 8).

### Antibody Responses to Truncated HRV-C VP1

To measure IgG1 binding to truncated HRV-C VP1, HRV-C3 VP1 was cloned and expressed from residue 14 to 275 in the pGEX-2T expression system. The purification protocol was the same as for full-length HRV-C3 VP1 and showed the same characteristics. CD analysis revealed similar structures to full-length HRV-C3 VP1, with the truncated HRV-C3 VP1 being composed of 33% β-strand and 12% α-helix (Table 2).

**Figure 9:**
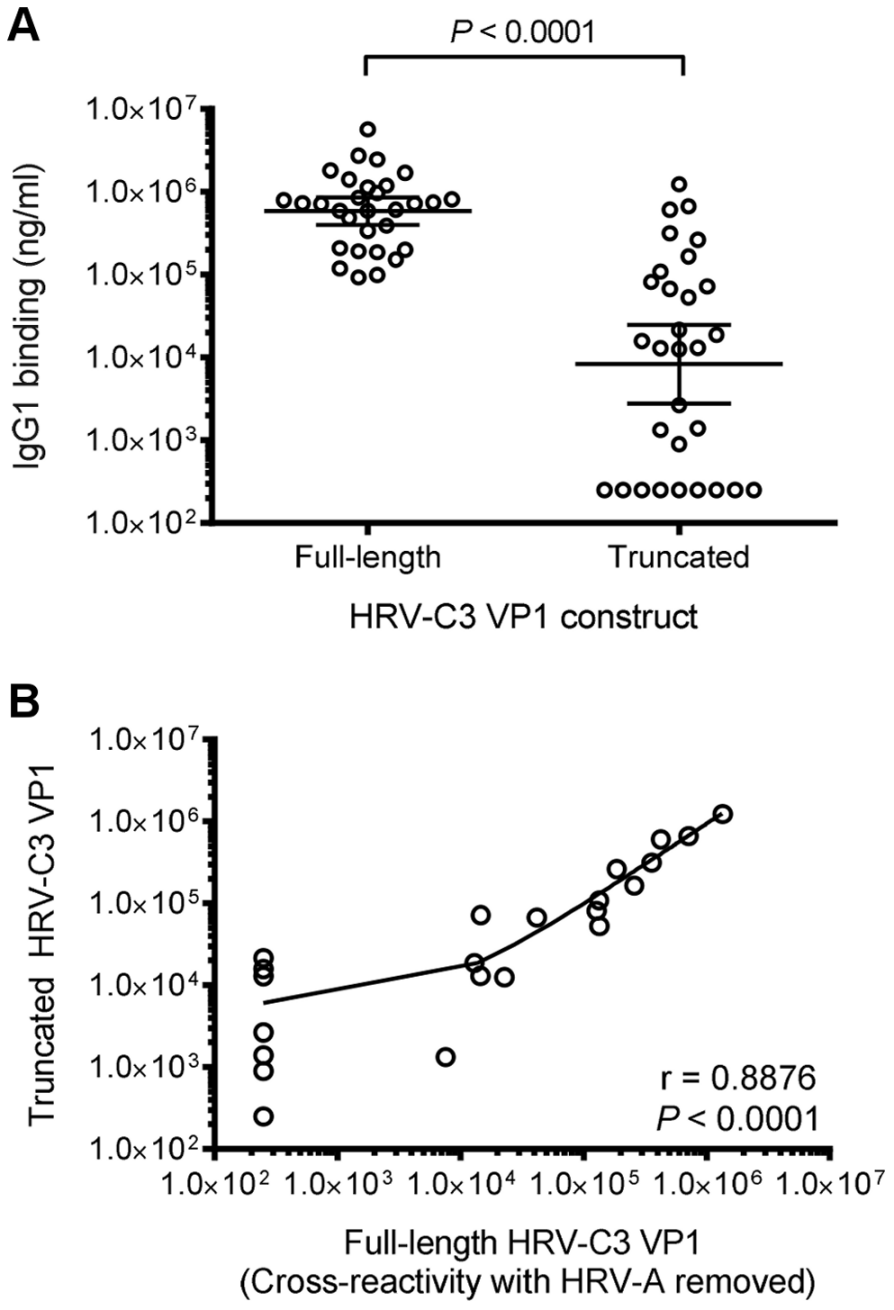
Antibody responses to HRV-C VP1 with an N-terminal truncation in 30 adult subjects. (A) IgG1 binding (ng/ml) to truncated HRV-C3 VP1 expressed from residue 14 to 275, in comparison to full-length VP1. *P*<0.0001 between full-length and truncated HRV-C3 VP1. (B) Correlation of IgG1 binding (ng/ml) between full-length HRV-C3 VP1 pre-absorbed with HRV-A to remove cross-reactivity to HRV-A, and truncated HRV-C3 VP1. A reference sera and three negative sera that did not have IgG1 binding to HRV-C3, as determined by the immunoassays and immunoabsorptions, were included on every plate for quantitation of binding.

IgG1 binding to truncated HRV-C3 VP1 was measured in 30 subjects who had high IgG1 titres to full-length HRV-C3 VP1. The IgG1 titres to truncated HRV-C3 VP1 were significantly lower than total IgG1 to full length VP1 (Figure 9A, *P*<0.0001) with 9/30 subjects having no reactivity to the truncated construct of HRV-C3 VP1. The sera with detectable antibody to truncated HRV-C3 VP1 were however, interestingly, the same as those with an antibody response to full-length HRV-C3 VP1 remaining after HRV-A absorption. There was a strong correlation between the titres of full-length HRV-C3 VP1 which had cross-reactivity with HRV-A species removed and the truncated construct (Figure 9B).

## Discussion

The VP1 antigen constructs of human rhinovirus species A, B and C all bound high titres of IgG1 antibody with negligible binding to myeloma controls. The total IgG1 binding by the HRV-A and -C antigens varied over a 2-log range but at high titre; the highest titres probably reflect antibodies induced by multiple infections with related viruses and recent infection causing transient elevations. The range of binding of specific titres at 250 - 1×10^7^ ng/ml is similar to values obtained for other microbial and vaccine antigens [24], [31]–[34]. All the antibody binding could be competitively inhibited with low concentrations of the homologous antigen. The recombinant glutathione-S-transferase VP1 fusion constructs purified by size exclusion chromatography were discrete, reproducible reagents that had a molecular mass equivalent to five VP1-GST dimers, which could reflect the pentameric interactions between the VP1 proteins in the apical pentameric subunits of the HRV particle [8], [35]. CD analysis of the HRV species showed that they had similar secondary structures to the natural protein.

The competitive inhibition performed with a sample of sera showed a sometimes high and variable degree of cross-reactivity between the different species including the HPV Sabin VP1, although as expected from the sequences, the competition was highest between HRV-A and -C. Given the variability between subjects observed in the pilot competitions, the assays were further developed by absorbing sera with an excess of the VP1 antigens from the heterologous HRV species and HPV Sabin and then conducting the titrations against the target VP1. The results showed a high prevalence of species-specific antibodies to HRV-A, HRV-B and HPV Sabin. It should be noted that the Sabin VP1 amino acid sequence (35% and 25% identical to HRV-A and C, respectively) can have nearly 60% identity to some Echoviruses, so this is an important consideration for future studies of HPV Sabin. The standout result here was the low prevalence of HRV-C specific titres even though most subjects had high total IgG1 antibody binding. The two genotypes examined for each HRV species gave the same results with high correlations between the species-specific titres that concurred with the competitive inhibition studies showing complete cross-competition with low doses of antigen. The pairs of genotypes that were selected to represent disparate sequences within each species (22%, 29% and 38% amino acid disparity within the HRV-A, -B and -C species, respectively) had the same species-specific binding especially for HRV-A, and where present, also to HRV-C. The response specific to an infecting genotype has not been measured in this study. Future studies could accomplish this by producing an antigen with the sequence of the known HRV genotype infecting the patient, and matching it to the patient anti-serum response. However, given the large shared IgG1 binding and the limited amino acid changes that alter neutralization [8], the antibodies against the infecting genotype might be difficult to measure, especially for HRV-A and HRV-B.

The low prevalence of species-specific binding to HRV-C is unlikely to be due to the poor antigenicity of the recombinant construct since it bound strongly prior to absorption with HRV-A and the other species, and could inhibit binding to itself with the same dose-response characteristics of HRV-A. The CD measurements also did not indicate a deficient structure or instability. Another anomaly is that, HRV-C infection is detectable at almost the same prevalence as HRV-A and twice that of HRV-B [36] for which species-specific antibody binding was far more prevalent. The cross-reactivity and sequence identity of HRV-C with HRV-A was greater than that of HRV-B but this does not account for the high prevalence of HRV-A specific binding after absorption with HRV-C. It is possible that for most subjects there is the phenomenon of “original antigenic sin” operating, where prior responses to HRV-A dictate the specificities induced by the related HRV-C and as described for influenza, can compromise the induction of protective responses [37]. HRV-C has also been reported to have greater genotypic diversity than HRV-A and HRV-B [38], although here the diverse genotypes correlated strongly. Speculatively, it might be due to the different biology perhaps with reduced growth due to its different receptor and unknown target cell and temperature requirements [39], [40]. A priority would be to determine if HRV-C infection does elicit HRV-C specific-antibodies and this would, given the low prevalence, might be easy to detect serology to monitor defined infectious episodes.

The degree of cross-reactivity between the HRV-A and HRV-C is not only unusual because of its unidirectional bias but is different in magnitude to that observed for many antigens with a similar degree of sequence disparity. Hyperimmune sera produced to serum albumins and haemoglobins from different species can readily distinguish between antigens with 70% sequence identity [41] and the anti-Psp-A antibodies to *Streptococcus pneumonia* with 69% sequence identity or less, induce largely non-cross-reactive responses [32]. The house dust mite allergens Der p 5 and Blo t 5 with 38% identity show no cross-reaction despite both frequently eliciting large IgE antibody responses [42].

The HRV-C construct made with the N-terminal truncation only detected IgG1 binding in the sera of subjects that retained anti-HRV-C antibodies when the cross-reactivity to HRV-A was removed by absorption. This could accordingly make a convenient construct for screening anti-HRV-C responses as the main cross-reactivity was with HRV-A, and minimal cross-reactivity with HRV-B or HPV Sabin was found. The truncation was originally studied because it was proposed that removing the surface inaccessible alpha helical region of the VP1 might prevent intermolecular interactions of the VP1 and improve the yields in recombinant expression. The same pentameric-sized complex was, however, produced and it retained similar structure to full-length HRV-C3 VP1. The N-terminal region has separately been of interest since it becomes exposed when the VP4 protein dissociates from the capsid in the acid environment of the lysosome [43]. Furthermore, a high frequency of IgG binding to a synthetic peptide representing this region has been detected [44]. There is sequence conservation between HRV-A and HRV-C within the truncated region that might explain the phenomenon but the truncation might also influence the conformation of other parts of VP1.

The study here describes recombinant constructs that can be produced in *E.coli* as structurally characterized, defined molecules to measure species-specific antibodies produced to HRV-A, -B and -C. The IgG binding studied to date shows evidence for a high degree of antibody production and for a high degree of cross-reactivity between the VP1 of different HRV species. Most notably, while some people have high titres of species-specific antibodies to HRV-C, most do not, even though they have high titres of cross-reactive antibodies mainly to HRV-A, that bind the HRV-C. Further investigation of these responses with clinically defined subjects and paediatric populations in conjunction with virus detection is thus warranted. The antibodies might be used as a marker of CD4 T-cell responses and be extended in the examination of other antibody isotypes. They might also have critical functions in the immunopathology produced by these viruses where for example they could increase the involvement of cytokine-producing monocytes and other inflammatory cells, as well as antigen presentation. As shown for other virus infections [12]–[14], non-neutralising antibodies also play a key role in immunity and they would be expected to be quantitatively a large component of early immune response to HRV immunity and to be influenced by prior infection by all species.

## Supporting Information

Figure S1 **Example of a standard curve used for quantitation of IgG1 binding.** Each assay was calibrated by interpolating the results from a titration curve constructed with recombinant GST-fusion Der p 2 and a standardised humanised anti-Der p 2 chimeric IgG1. The linear section of the Der p 2 chimeric curve used for analysis had a slope = 5.00±0.07 and is indicated in red. Equivalent concentrations of recombinant VP1 antigens was used to coat wells and bound with a titration of reference sera (example of HRV-A34 shown). The 2-fold dilutions used are indicated. The linear section of the reference titration curve (indicated in red) had a slope = 4.95±0.69, comparable to the Der p 2 chimeric curve. A titration curve of reference sera was included on every plate to construct a standard curve, which was then used to convert europium counts to absolute IgG1 binding (ng/ml). The mean europium count of the negative control, human myeloma IgG1, is indicated. For HRV-A34, the mean and standard deviation (SD) europium count for human myeloma IgG1 was 2572±795 (n = 3), which was calculated to be 499 ng/ml. Negative sera was determined using mean +3SD of human myeloma IgG1 binding for each antigen.
(TIF)

Figure S2 **Circular dichroism (CD) analysis of recombinant HRV-C VP1 protein.** (A) CD spectrum of GST-fusion HRV-C3 VP1. (B) CD spectrum of HRV-C3 VP1 following subtractive CD analysis in which the GST control was subtracted from the fusion protein. The diagrams represent the ultraviolet spectra of the purified recombinant proteins analysed using CD spectroscopy in the range 260–190 nm. Structural analysis of the data was performed using DiChroWeb Server: CDSSTR algorithm, reference set 4.
(TIF)

Table S1 **Nucleotide and protein sequences of HRV and HPV Sabin VP1 used in this study.**
(DOCX)
