# Supplementary figures and images for: Species-Specific and Cross-Reactive IgG1 Antibody Binding to Viral Capsid Protein 1 (VP1) Antigens of Human Rhinovirus Species A, B and C
Source: PLoS One. 2013 Aug 7;8(8):e70552. doi: 10.1371/journal.pone.0070552 (PMC3737412; doi:10.1371/journal.pone.0070552)

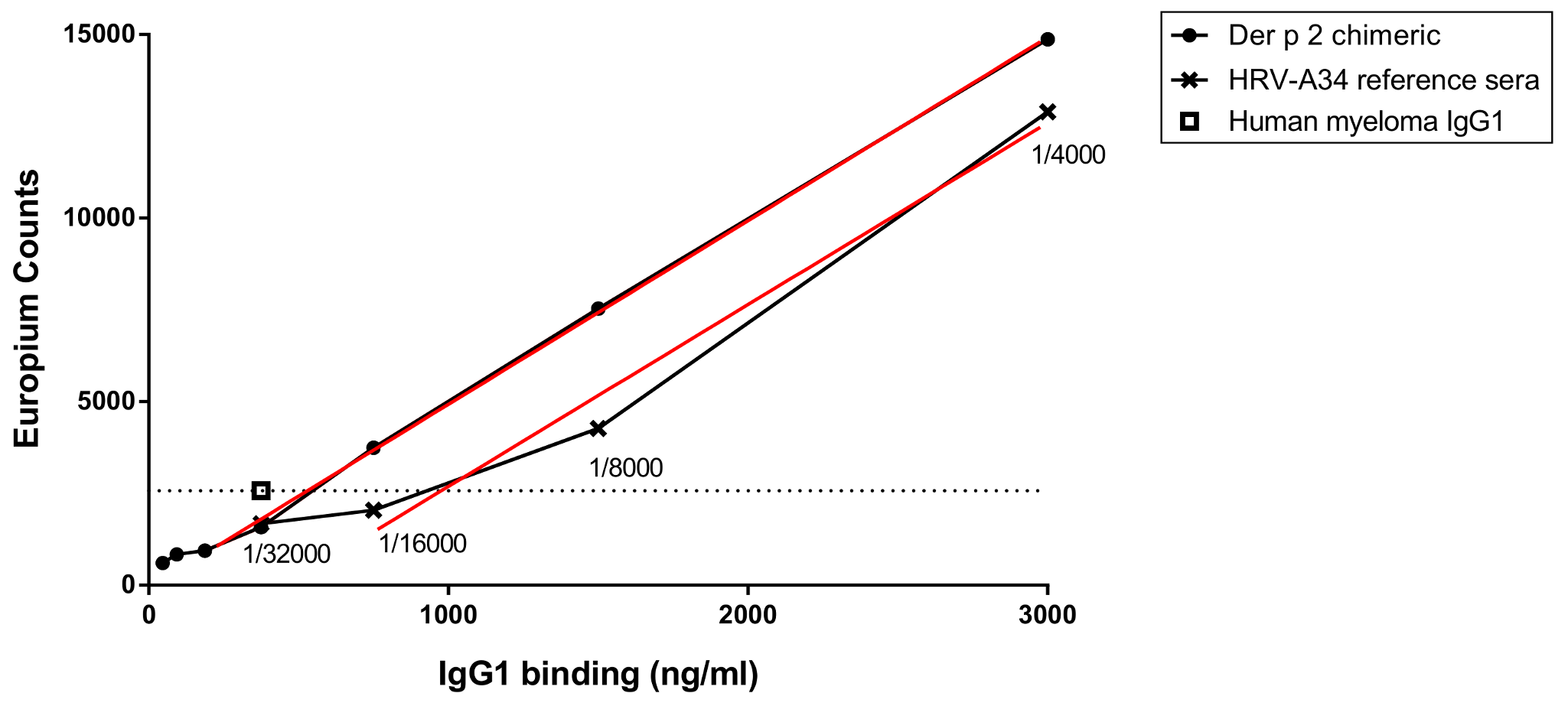

Supplement: Figure S1 — Example of a standard curve used for quantitation of IgG1 binding. Each assay was calibrated by interpolating the results from a titration curve constructed with recombinant GST-fusion Der p 2 and a standardised humanised anti-Der p 2 chimeric IgG1. The linear section of the Der p 2 chimeric curve used for analysis had a slope = 5.00±0.07 and is indicated in red. Equivalent concentrations of recombinant VP1 antigens was used to coat wells and bound with a titration of reference sera (example of HRV-A34 shown). The 2-fold dilutions used are indicated. The linear section of the reference titration curve (indicated in red) had a slope = 4.95±0.69, comparable to the Der p 2 chimeric curve. A titration curve of reference sera was included on every plate to construct a standard curve, which was then used to convert europium counts to absolute IgG1 binding (ng/ml). The mean europium count of the negative control, human myeloma IgG1, is indicated. For HRV-A34, the mean and standard deviation (SD) europium count for human myeloma IgG1 was 2572±795 (n = 3), which was calculated to be 499 ng/ml. Negative sera was determined using mean +3SD of human myeloma IgG1 binding for each antigen. (TIF) [file pone.0070552.s001.tif]

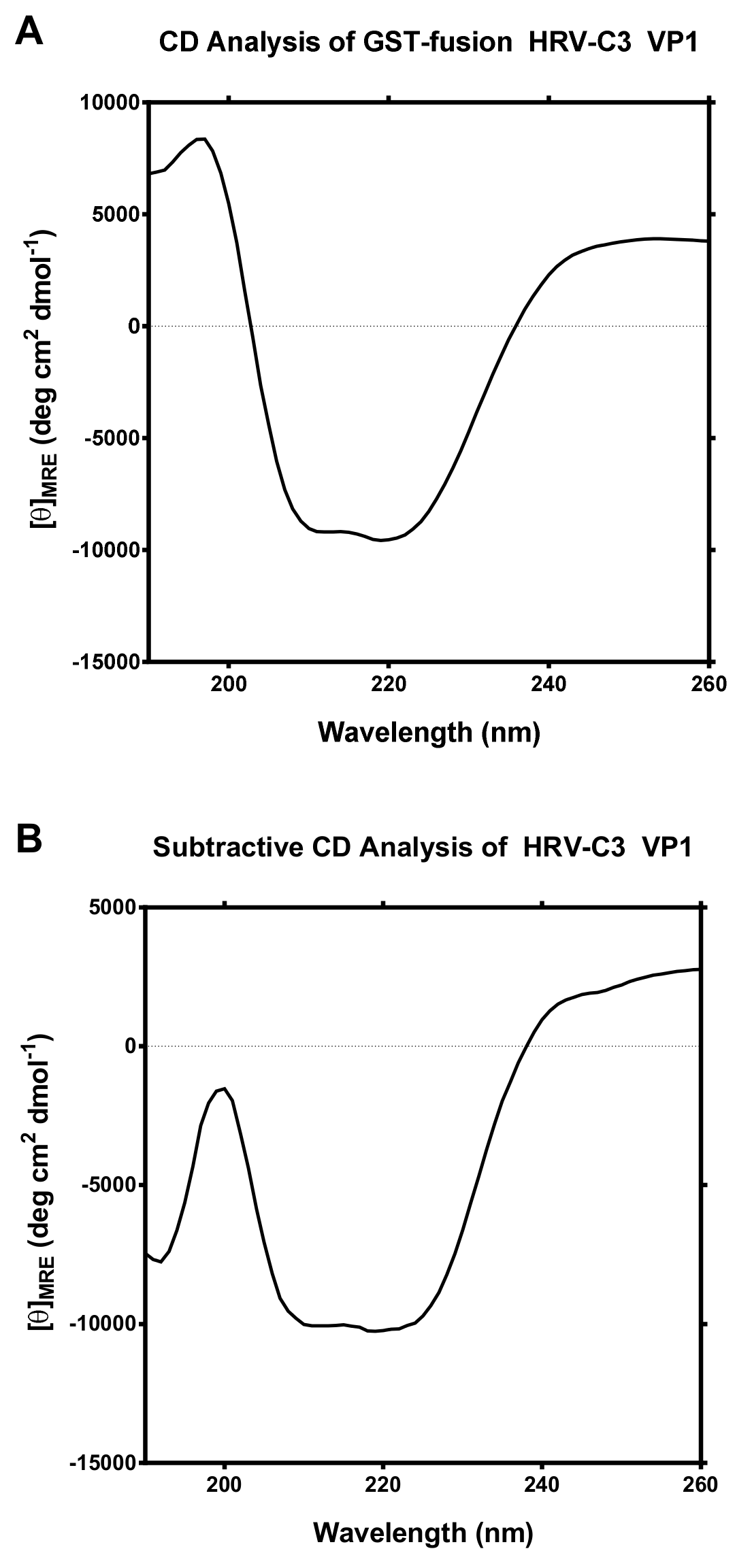

Supplement: Figure S2 — Circular dichroism (CD) analysis of recombinant HRV-C VP1 protein. (A) CD spectrum of GST-fusion HRV-C3 VP1. (B) CD spectrum of HRV-C3 VP1 following subtractive CD analysis in which the GST control was subtracted from the fusion protein. The diagrams represent the ultraviolet spectra of the purified recombinant proteins analysed using CD spectroscopy in the range 260–190 nm. Structural analysis of the data was performed using DiChroWeb Server: CDSSTR algorithm, reference set 4. (TIF) [file pone.0070552.s002.tif]
