# Supplementary material for: Species-Specific and Cross-Reactive IgG1 Antibody Binding to Viral Capsid Protein 1 (VP1) Antigens of Human Rhinovirus Species A, B and C
Source: PLoS One. 2013 Aug 7;8(8):e70552. doi: 10.1371/journal.pone.0070552 (PMC3737412; doi:10.1371/journal.pone.0070552)
Supplement: Table S1 — Nucleotide and protein sequences of HRV and HPV Sabin VP1 used in this study. (DOCX) [file pone.0070552.s003.docx]

| **Name** | **Accession number** | **Optimized nucleotide sequence (for expression in *E.coli*)*** | **Protein sequence** |
| --- | --- | --- | --- |
| HRV-A34 VP1 | FJ445189.1 | AACCCGGTCGAAAACTACATTGATGAAGTGCTGAACGAAGTGCTGGTCGTCCCGAACATTAAAGAATCGCAGGCTACCACCTCCAACTCAGCCCCGGCACTGGATGCCGCGGAAACCGGTCATACGAGCTCTGTGCAGCCGGAAGACATGATTGAAACCCGTTATGTTCAGACCTCCCAAACGCGCGATGAAATGAGTATTGAATCCTTTCTGGGCCGTAGTGGTTGCATCCACATGTCCAAACTGGTGGTTGATTACGAAAACTACAACGCGAAAACCAAAAACTTCATGACGTGGCAGATTAATCTGCAAGAAATGGCCCAGATCCGTCGCAAATTTGAAATGTTCACCTACGTGCGCTTCGATTCAGAAGTGACGCTGGTTCCGTCGATTGCAGCTAAAGGCGATGACATCGGTCATGTCGTGATGCAATATATGTACGTTCCGCCGGGTGCGCCGATTCCGAAAACCCGTGATGACTTTGCGTGGCAGAGCGGCACGAACGCCTCTATCTTCTGGCAGCACGGTCAAACCTATCCGCGCTTTAGCCTGCCGTTCCTGTCAATCGCATCGGCTTATTACATGTTCTATGATGGCTACGATGGTGACCAACATGACAGCCGTTACGGCACCGTTGTCACGAACGATATGGGTACCCTGTGCTCTCGCATTGTCACGGACGAACACCAGAATCGTGTCGAAATCACCACGCGCGTGTATCATAAAGCAAAACACGTTAAAACCTGGTGTCCGCGTCCGCCGCGCGCTGTTGAATATACCCATACGCACGTCACCAATTACAAAGTCCGTGGCAAAACCGAAAAAACCGCTATCAAACACCGTGCGAAAATTACGATGGCG | NPVENYIDEVLNEVLVVPNIKESQATTSNSAPALDAAETGHTSSVQPEDMIETRYVQTSQTRDEMSIESFLGRSGCIHMSKLVVDYENYNAKTKNFMTWQINLQEMAQIRRKFEMFTYVRFDSEVTLVPSIAAKGDDIGHVVMQYMYVPPGAPIPKTRDDFAWQSGTNASIFWQHGQTYPRFSLPFLSIASAYYMFYDGYDGDQHDSRYGTVVTNDMGTLCSRIVTDEHQNRVEITTRVYHKAKHVKTWCPRPPRAVEYTHTHVTNYKVRGKTEKTAIKHRAKITMA |
| HRV-A1B VP1 | D00239.1 | AACCCGGTGGAAAATTATATTGATGAAGTGCTGAACGAAGTTCTGGT  GGTTCCGAATATCAAAGAAAGCCATCACACCACGAGCAACTCTGCAC  CGCTGCTGGATGCAGCAGAAACCGGTCATACGAGTAATGTGCAGCCG  GAAGATGCGATTGAAACCCGTTACGTTATGACCAGCCAGACGCGCGA  TGAAATGAGTATCGAAAGCTTTCTGGGCCGTAGTGGTTGCGTGCACATTAGCCGCATCAAAGTTGATTACAACGATTACAACGGTGTGAACAAA  AACTTCACCACGTGGAAAATCACCCTGCAGGAAATGGCGCAGATCCG  TCGCAAATTTGAACTGTTCACCTATGTTCGTTTCGATAGCGAAGTGAC  GCTGGTTCCGTGTATTGCCGGCCGCGGTGATGATATCGGCCATGTGGT  TATGCAGTATATGTACGTGCCGCCGGGTGCACCGATTCCGAAAACCC  GTAACGATTTTTCTTGGCAGAGTGGCACGAATATGAGCATCTTCTGGC  AGCACGGTCAGCCGTTTCCGCGCTTCTCTCTGCCGTTTCTGAGCATTG  CATCTGCGTATTACATGTTCTATGATGGCTACGATGGTGATAACAGCT  CTAGTAAATATGGCTCTATCGTGACCAATGATATGGGTACGATTTGCA  GTCGTATCGTTACCGAAAAACAGGAACATCCGGTGGTTATTACCACG  CATATCTACCACAAAGCCAAACACACCAAAGCATGGTGTCCGCGTCC  GCCGCGCGCCGTGCCGTATACCCATTCTCGTGTGACGAACTACGTTCC  GAAAACCGGCGATGTGACCACGGCGATTGTTCCGCGTGCGAGCATGA  AAACCGTT | NPVENYIDEVLNEVLVVPNIKESHHTTSNSAPLLDAAETGHTSNVQPEDAIETRYVMTSQTRDEMSIESFLGRSGCVHISRIKVDYNDYNGVNKNFTTWKITLQEMAQIRRKFELFTYVRFDSEVTLVPCIAGRGDDIGHVVMQYMYVPPGAPIPKTRNDFSWQSGTNMSIFWQHGQPFPRFSLPFLSIASAYYMFYDGYDGDNSSSKYGSIVTNDMGTICSRIVTEKQEHPVVITTHIYHKAKHTKAWCPRPPRAVPYTHSRVTNYVPKTGDVTTAIVPRASMKTV |
| HRV-B14 VP1 | NC001490 | GGTCTGGGTGACGAACTGGAAGAAGTGATTGTGGAAAAAACGAAACAAACGGTTGCCTCTATCTCATCTGGTCCGAAACATACGCAGAAAGTTCCGATCCTGACCGCGAACGAAACCGGTGCAACGATGCCGGTCCTGCCGTCAGATTCGATTGAAACCCGTACCACGTATATGCATTTTAATGGCTCCGAAACGGACGTGGAATGCTTCCTGGGTCGCGCGGCCTGTGTCCATGTGACCGAAATTCAGAACAAAGATGCAACGGGCATCGACAATCACCGTGAAGCTAAACTGTTCAACGATTGGAAAATCAACCTGAGCTCTCTGGTGCAACTGCGTAAAAAACTGGAACTGTTTACCTATGTTCGCTTCGATAGCGAATACACCATTCTGGCAACGGCTTCACAGCCGGACTCGGCGAACTATAGTTCCAATCTGGTGGTTCAAGCGATGTACGTTCCGCATGGCGCCCCGAAAAGTAAACGTGTCGGTGATTACACCTGGCAGAGCGCCTCTAACCCGTCCGTGTTTTTCAAAGTTGGCGACACGAGTCGCTTTTCCGTTCCGTATGTCGGTCTGGCGAGCGCCTACAATTGCTTCTATGATGGCTACTCTCATGATGACGCCGAAACCCAATATGGCATTACGGTCCTGAACCACATGGGTAGCATGGCATTCCGCATCGTGAATGAACATGACGAACACAAAACCCTGGTCAAAATTCGTGTGTACCACCGCGCAAAACTGGTGGAAGCTTGGATTCCGCGTGCACCGCGTGCTCTGCCGTATACCTCAATCGGTCGTACGAACTACCCGAAAAATACCGAACCGGTTATTAAAAAACGCAAAGGCGATATCAAATCGTAT | GLGDELEEVIVEKTKQTVASISSGPKHTQKVPILTANETGATMPVLPSDSIETRTTYMHFNGSETDVECFLGRAACVHVTEIQNKDATGIDNHREAKLFNDWKINLSSLVQLRKKLELFTYVRFDSEYTILATASQPDSANYSSNLVVQAMYVPHGAPKSKRVGDYTWQSASNPSVFFKVGDTSRFSVPYVGLASAYNCFYDGYSHDDAETQYGITVLNHMGSMAFRIVNEHDEHKTLVKIRVYHRAKLVEAWIPRAPRALPYTSIGRTNYPKNTEPVIKKRKGDIKSY |
| HRV-B69 VP1 | FJ445151 | GGTCTGGGCGAAGAACTGGAAGAAGTCGTTATTGACAAAATGAAACAAGTGACGGCATCGGTGCAAAGCGGTAGCAAACACACGCAGAAAGTTCCGGCGCTGAGTGCCTCCGAAACCGGTGCAACGCTGCCGACCAACCCGTCAGATTCGGTCGAAACCCGTACCACGTATATGCATTTTACGGGCAGTGAAACCACGATTGAAAACTTCCTGGGTCGCTCCGCTTGCGTGCACATTACCGAAATCCAGAATAAAAAACCGATCGAAAGCCTGGAAGAAGGCAAAAACACGCATAAGGGTCAGATGCTGTTTAATGATTGGAAAATTTCTCTGAGCTCTCTGGTCCAACTGCGTAAAAAACTGGAACTGTTTACCTATGTGCGCTTCGACAGCGAATACACGATCCTGGCGACCGCCTCTCAGCCGAACGCAAGTGAATATGCTTCCAATCTGACCGTGCAAGCAATGTACGTTCCGCCGGGTGCTCCGAACCCGGTGAAATGGAATGATTATACCTGGCAGAGCGCGTCTAATCCGAGCGTCTTTTTCGAAGTGGGCAAAACCGCCCGTTTTTCAGTTCCGTTCACGGGTATTGCATCGGCTTATAACTGTTTTTATGACGGCTACTCACATGATAATGAAGACACCCAATACGGCATTAACGTCCTGAATCACATGGGTTCGATCGCCTTTCGCGTGGTTAACGATCATGACGAACACACCACGATTGTTAAAATCCGTGTCTTCCATCGCGCGAAACACATTCGTGCATGGATTCCGCGTCCGCCGCGTGCACTGCCGTATACGAGCATCGGCCGCACCAACTTCCCGAATACGCACGGTAAAGTTATTACCCGTCGCAATAAAATCACCACGTAC | GLGEELEEVVIDKMKQVTASVQSGSKHTQKVPALSASETGATLPTNPSDSVETRTTYMHFTGSETTIENFLGRSACVHITEIQNKKPIESLEEGKNTHKGQMLFNDWKISLSSLVQLRKKLELFTYVRFDSEYTILATASQPNASEYASNLTVQAMYVPPGAPNPVKWNDYTWQSASNPSVFFEVGKTARFSVPFTGIASAYNCFYDGYSHDNEDTQYGINVLNHMGSIAFRVVNDHDEHTTIVKIRVFHRAKHIRAWIPRPPRALPYTSIGRTNFPNTHGKVITRRNKITTY |
| HRV-C3 VP1 | EF186077 | AATCCGGTGGAAGAATTTGTTGAACATACCCTGAAAGAAGTTCTGGTGGTGCCGGATACGCAGGCATCTGGTCCGGTTCACACCACGAAACCGCAGGCACTGGGTGCAGTGGAAATTGGTGCAACCGCGGATGTTGGTCCGGAAACCCTGATCGAAACGCGTTATGTGATGAACGATAATACCAACGCAGAAGCGGCCGTTGAAAATTTTCTGGGTCGTAGCGCACTGTGGGCAAACCTGCGTCTGGATCAGGGTTTTCGCAAATGGGAAATTAACTTCCAGGAACATGCGCAAGTGCGTAAAAAATTTGAAATGTTCACCTACGTGCGCTTTGATCTGGAAATTACCATCGTTACGAACAATAAAGGCCTGATGCAGATTATGTTCGTGCCGCCGGGTATCACCCCGCCGGGCGGTAAAGATGGTCGTGAATGGGATACGGCGAGTAATCCGAGCGTGTTTTTCCAGCCGAACTCTGGTTTTCCGCGCTTCACCATTCCGTTTACGGGCCTGGGTAGTGCGTATTACATGTTCTATGATGGCTACGATGGTACCGATGATGCCAATATTAACTATGGCATCAGCCTGACCAATGATATGGGTACGCTGTGCTTTCGTGCACTGGATGGCACCGGTGCGTCTGATATCAAAGTTTTCGGCAAACCGAAACATATTACCGCATGGATTCCGCGTCCGCCGCGCGCAACCCAGTATCTGCACAAATTCAGTACGAACTACAACAAACCGAAAACCAGCGGTTCTACGGAACTGGAACCGAAACACTTTTTCAAATACCGCCAGGATATTACCAGCATCACGAACCTG | NPVEEFVEHTLKEVLVVPDTQASGPVHTTKPQALGAVEIGATADVGPETLIETRYVMNDNTNAEAAVENFLGRSALWANLRLDQGFRKWEINFQEHAQVRKKFEMFTYVRFDLEITIVTNNKGLMQIMFVPPGITPPGGKDGREWDTASNPSVFFQPNSGFPRFTIPFTGLGSAYYMFYDGYDGTDDANINYGISLTNDMGTLCFRALDGTGASDIKVFGKPKHITAWIPRPPRATQYLHKFSTNYNKPKTSGSTELEPKHFFKYRQDITSITNL |
| HRV-C5 VP1 | EF582386 | AACCCGGTGGAACAGTTTGTGGATAATGTTCTGGAAGAAGTTCTGGTGGTTCCGAACACGCAGCCGAGCGGTCCGATTCATACCACGAAACCGACCGCGCTGTCTGCGATGGAAATTGGTGCGAGCTCTGATGTGAAACCGGAAGATATGATCGAAACCCGTTATGTGGTTAACAGTCGCACGAATGGCGAAGCCACCATTGAAAATTTCCTGGGTCGTAGCGCACTGTGGGCGAACATCGATATGGCCAATGGCTATGCAACGTGGTCTATTACCTACCAGGGTAACGCACAGATCCGTAAAAAACTGGAACTGTTTACCTATGTGCGCTTCGATCTGGAAATTACCATTATCACGAGTAATAGTAGCCTGATGCAGATCATGTACGTTCCGCCGGGCGCCAAACCGCCAAACAATAACTCTAGTATGGAATGGAACACCGCAAGCAATCCGTCTATTTTCTTTCAGCCGGGCAACGGTTTTCCGCGCTTCACGATCCCGTTTACCGGCCTGGGTAGCGCCTATTACATGTTCTATGATGGCTACGATAAAGTGTCTCATGATGAAGGCACCTATGGTACGAGTGTTACCAATGATATGGGTCGTCTGTGCTTTCGCACGCCGAATAACAATAGCGGCACCGATATTATCCGTATTTTCGGTAAACCGAAACATACCCGCGCATGGATTCCGCGTCCGCCGCGCGCGACGGAATATACCCACAAAGATTCTACCAACTACAATCGTCGCGTGAACCCGAATAGTGAAGATAGCACCCTGACGCGTGATCACTACATTAAAACGCGCGCGACCGTTACCACGGCC | NPVEQFVDNVLEEVLVVPNTQPSGPIHTTKPTALSAMEIGASSDVKPEDMIETRYVVNSRTNGEATIENFLGRSALWANIDMANGYATWSITYQGNAQIRKKLELFTYVRFDLEITIITSNSSLMQIMYVPPGAKPPNNNSSMEWNTASNPSIFFQPGNGFPRFTIPFTGLGSAYYMFYDGYDKVSHDEGTYGTSVTNDMGRLCFRTPNNNSGTDIIRIFGKPKHTRAWIPRPPRATEYTHKDSTNYNRRVNPNSEDSTLTRDHYIKTRATVTTA |
| HPV Sabin VP1 | AY184219.1 | GGCCTGGGTCAGATGCTGGAAAGTATGATTGATAACACCGTGCGTGAAACGGTTGGTGCAGCAACCAGCCGCGATGCACTGCCGAATACCGAAGCGTCTGGTCCGGCCCATAGTAAAGAAATCCCGGCACTGACCGCAGTTGAAACCGGTGCAACGAACCCGCTGGTGCCGTCTGATACCGTTCAGACGCGTCATGTGGTTCAGCACCGTTCTCGCAGTGAAAGCTCTATTGAATCTTTCTTTGCGCGCGGTGCCTGCGTGGCAATTATCACCGTTGATAACAGCGCATCTACGAAAAACAAAGATAAACTGTTCACCGTTTGGAAAATCACCTATAAAGATACGGTGCAGCTGCGTCGCAAACTGGAATTTTTCACCTACAGCCGTTTTGATATGGAATTTACCTTCGTGGTTACGGCAAACTTCACCGAAACGAACAATGGCCATGCGCTGAATCAGGTGTATCAGATTATGTACGTTCCGCCGGGTGCACCGGTGCCGGAAAAATGGGATGATTATACCTGGCAGACGAGTAGCAACCCGAGCATCTTTTATACCTACGGTACGGCACCGGCACGTATTTCTGTGCCGTACGTTGGCATCAGTAATGCGTATAGCCACTTTTACGATGGTTTCAGCAAAGTGCCGCTGAAAGATCAGAGCGCAGCGCTGGGCGATTCTCTGTATGGTGCCGCAAGTCTGAATGATTTCGGTATTCTGGCCGTTCGCGTGGTTAACGATCATAATCCGACCAAAGTTACGTCTAAAATTCGTGTGTATCTGAAACCGAAACACATCCGTGTGTGGTGTCCGCGTCCGCCGCGCGCAGTTGCATATTACGGTCCGGGTGTGGATTATAAAGATGGCACCCTGACGCCGCTGAGCACCAAAGATCTGACCACGTAC | GLGQMLESMIDNTVRETVGAATSRDALPNTEASGPAHSKEIPALTAVETGATNPLVPSDTVQTRHVVQHRSRSESSIESFFARGACVAIITVDNSASTKNKDKLFTVWKITYKDTVQLRRKLEFFTYSRFDMEFTFVVTANFTETNNGHALNQVYQIMYVPPGAPVPEKWDDYTWQTSSNPSIFYTYGTAPARISVPYVGISNAYSHFYDGFSKVPLKDQSAALGDSLYGAASLNDFGILAVRVVNDHNPTKVTSKIRVYLKPKHIRVWCPRPPRAVAYYGPGVDYKDGTLTPLSTKDLTTY |

*Codons that were changed from the published nucleotide sequence for optimization for expression in *E.coli* are highlighted in red
